# Supplementary material for: A Likelihood Approach for Real-Time Calibration of Stochastic Compartmental Epidemic Models
Source: PLoS Comput Biol. 2017 Jan 17;13(1):e1005257. doi: 10.1371/journal.pcbi.1005257 (PMC5240920; doi:10.1371/journal.pcbi.1005257)
Supplement: S1 File — (TAR.GZ) [file pcbi.1005257.s014.tar.gz › HSPH_Online-SI-Revision/Additions/MSS11/n100-extreme-SEITR_noisy/n100-extreme-SEITR_noisy_NEW2_table-4.pdf]

1	newly 3 weeks spec	13 577.1	9321.09	{9158., 9471.}	31.3467	0.163372	newly 3 weeks cumul	31 325.2	22 600.5	{22 300., 22 904.}	27.8519	0.141799	newly 5 weeks spec	12 047.4	9576.66	{9399., 9745.}	20.5082	0.0997159	newly 5 weeks cumul	57 070.8	42 144.1	{41 712., 42 570.}	26.1546	0.131691	newly 54 weeks cumul	86 564.8	81 662.9	{81 437., 81 890.}	5.6627	0.0253175
2	newly 3 weeks spec	17 235.	12 041.9	{7939., 14 267.}	30.1313	0.165418	newly 3 weeks cumul	29 177.	21 185.4	{13 662., 25 548.}	27.39	0.150046	newly 5 weeks spec	17 453.	15 200.7	{13 153., 15 927.}	12.9052	0.0613741	newly 5 weeks cumul	67 910.	52 259.6	{37 320., 58 458.}	23.0458	0.118404	newly 62 weeks cumul	97 717.	84 278.	{69 812., 90 438.}	13.753	0.0669693
3	newly 3 weeks spec	14 424.	18 529.9	{16 211., 20 617.}	28.6554	0.107487	newly 3 weeks cumul	24 222.	31 390.7	{26 476., 35 380.}	30.0959	0.111935	newly 5 weeks spec	18 266.	13 581.1	{9949., 15 697.}	25.6483	0.134427	newly 5 weeks cumul	61 841.	64 772.	{58 113., 71 672.}	7.86861	0.0326592	newly 64 weeks cumul	97 783.	79 351.6	{65 807., 91 271.}	18.8493	0.0936318
4	newly 3 weeks spec	13 822.	16 775.	{16 534., 17 222.}	21.3646	0.0838312	newly 3 weeks cumul	25 848.	32 223.5	{31 324., 33 199.}	24.6654	0.0953867	newly 5 weeks spec	16 022.	13 993.3	{13 675., 14 286.}	12.662	0.0588641	newly 5 weeks cumul	59 339.	63 890.5	{63 310., 64 812.}	7.9259	0.0331068	newly 62 weeks cumul	94 062.	87 790.1	{87 765., 88 263.}	6.66785	0.0300847
5	newly 3 weeks spec	22 578.	18 167.4	{14 896., 20 797.}	19.535	0.098067	newly 3 weeks cumul	36 477.	30 431.7	{23 897., 35 280.}	16.5746	0.0839764	newly 5 weeks spec	16 920.	14 262.	{11 402., 16 113.}	16.7238	0.0831622	newly 5 weeks cumul	77 798.	64 744.9	{58 144., 72 080.}	16.7782	0.0812547	newly 64 weeks cumul	97 923.	80 580.7	{66 439., 91 333.}	17.7101	0.0870562
6	newly 3 weeks spec	20 143.	17 111.4	{15 694., 18 219.}	15.0506	0.0715408	newly 3 weeks cumul	34 123.	29 995.3	{26 782., 32 685.}	12.0965	0.0571873	newly 5 weeks spec	17 405.	15 543.6	{14 982., 16 138.}	10.6946	0.0493081	newly 5 weeks cumul	74 866.	64 900.2	{60 617., 67 180.}	13.3115	0.062297	newly 63 weeks cumul	93 450.	90 442.1	{81 952., 92 556.}	3.21872	0.0146545
7	newly 3 weeks spec	12 108.	10 027.3	{9753., 10 231.}	17.1848	0.0819593	newly 3 weeks cumul	28 975.	25 086.4	{24 547., 25 578.}	13.4206	0.0626378	newly 5 weeks spec	11 332.	9017.44	{8852., 9200.}	20.425	0.0992709	newly 5 weeks cumul	52 923.	44 195.4	{43 497., 44 888.}	16.4911	0.0782945	newly 55 weeks cumul	88 800.	70 811.4	{70 444., 71 154.}	20.2575	0.0983126
8	newly 3 weeks spec	16 980.	16 015.4	{14 391., 17 179.}	6.42279	0.0295626	newly 3 weeks cumul	32 632.	30 354.8	{26 637., 33 385.}	8.02476	0.0373972	newly 5 weeks spec	14 766.	14 030.3	{13 306., 14 733.}	5.14682	0.0232073	newly 5 weeks cumul	65 812.	61 771.7	{56 656., 64 734.}	6.14803	0.0281238	newly 62 weeks cumul	97 109.	85 690.1	{77 082., 90 405.}	11.7589	0.0552243
9	newly 3 weeks spec	23 304.	8143.92	{2657., 11 315.}	65.0536	0.5734	newly 3 weeks cumul	35 304.	12 103.7	{4028., 16 875.}	65.7159	0.574249	newly 5 weeks spec	17 411.	17 269.	{12 251., 19 593.}	16.4997	0.151913	newly 5 weeks cumul	78 660.	44 149.7	{22 670., 54 606.}	43.8728	0.344317	newly 64 weeks cumul	98 833.	87 382.8	{79 017., 93 572.}	11.5854	0.0559684
10	newly 3 weeks spec	12 030.	11 871.3	{11 252., 12 789.}	3.86018	0.0169096	newly 3 weeks cumul	25 011.	24 542.2	{23 279., 25 773.}	3.43821	0.0152282	newly 5 weeks spec	13 614.	11 325.4	{10 543., 13 422.}	16.83	0.0818321	newly 5 weeks cumul	52 825.	48 922.	{46 579., 53 778.}	8.11373	0.036992	newly 60 weeks cumul	95 503.	71 544.4	{66 853., 87 847.}	25.0868	0.128058
11	newly 3 weeks spec	13 896.	9441.05	{8324., 9975.}	32.0592	0.168743	newly 3 weeks cumul	26 326.	18 516.7	{16 142., 19 571.}	29.664	0.153872	newly 5 weeks spec	14 675.	12 770.2	{12 422., 12 976.}	12.98	0.0604629	newly 5 weeks cumul	57 151.	43 447.	{39 948., 44 820.}	23.9786	0.119507	newly 59 weeks cumul	97 210.	87 495.6	{81 180., 91 229.}	9.99325	0.0461289
12	newly 3 weeks spec	16 108.	13 562.5	{11 668., 14 121.}	15.8026	0.0759004	newly 3 weeks cumul	33 166.	28 927.6	{24 342., 30 405.}	12.7794	0.0605516	newly 5 weeks spec	14 159.	11 634.8	{10 983., 12 126.}	17.8279	0.0868922	newly 5 weeks cumul	64 453.	54 648.8	{47 967., 56 617.}	15.2113	0.0727921	newly 60 weeks cumul	95 054.	76 565.6	{67 542., 78 964.}	19.4504	0.0955384
13	newly 3 weeks spec	15 592.	13 867.3	{11 224., 14 800.}	11.0614	0.0529465	newly 3 weeks cumul	30 732.	27 231.3	{22 757., 29 206.}	11.3911	0.0542859	newly 5 weeks spec	14 704.	13 304.2	{9741., 14 153.}	9.51965	0.0465134	newly 5 weeks cumul	62 511.	55 962.4	{44 684., 59 205.}	10.476	0.0500783	newly 61 weeks cumul	96 828.	84 196.6	{58 284., 91 345.}	13.0452	0.0651245
14	newly 3 weeks spec	10 081.	8669.2	{8474., 8872.}	14.0046	0.065605	newly 3 weeks cumul	23 444.	20 702.3	{20 387., 21 123.}	11.6946	0.0541004	newly 5 weeks spec	11 642.	9227.6	{9037., 9428.}	20.7387	0.100989	newly 5 weeks cumul	46 832.	39 384.7	{38 821., 39 985.}	15.9023	0.0752557	newly 54 weeks cumul	89 533.	74 741.4	{74 394., 75 053.}	16.5208	0.0784252
15	newly 3 weeks spec	14 236.	8855.78	{8673., 9034.}	37.7931	0.206211	newly 3 weeks cumul	30 841.	20 267.5	{19 908., 20 637.}	34.2838	0.182372	newly 5 weeks spec	13 230.	10 125.4	{9954., 10 286.}	23.4664	0.116179	newly 5 weeks cumul	59 486.	40 472.8	{40 022., 40 945.}	31.9624	0.167266	newly 55 weeks cumul	91 749.	85 828.7	{82 678., 87 665.}	6.45271	0.029157
16	newly 3 weeks spec	11 254.	10 428.9	{5677., 12 422.}	15.0661	0.0819777	newly 3 weeks cumul	24 092.	23 802.3	{15 397., 28 110.}	14.7883	0.0706114	newly 5 weeks spec	12 953.	9740.54	{3767., 11 545.}	24.8009	0.159271	newly 5 weeks cumul	50 447.	44 486.4	{24 277., 52 491.}	14.5245	0.0832996	newly 58 weeks cumul	87 287.	70 031.8	{30 712., 82 820.}	19.8823	0.125336
17	newly 3 weeks spec	15 615.	17 590.8	{13 644., 20 838.}	18.6391	0.0743301	newly 3 weeks cumul	24 517.	28 970.1	{21 035., 35 102.}	24.7924	0.0959541	newly 5 weeks spec	20 574.	15 295.9	{13 293., 17 789.}	25.6545	0.131767	newly 5 weeks cumul	68 260.	64 672.6	{58 693., 71 783.}	8.73494	0.0398237	newly 64 weeks cumul	92 364.	83 005.7	{75 150., 91 368.}	10.1319	0.0480484
18	newly 3 weeks spec	19 007.	9705.52	{7410., 13 240.}	48.9371	0.303718	newly 3 weeks cumul	31 170.	15 534.7	{11 610., 21 490.}	50.1613	0.315404	newly 5 weeks spec	17 189.	17 045.6	{16 237., 17 884.}	3.93967	0.0181349	newly 5 weeks cumul	70 235.	48 107.7	{41 973., 57 753.}	31.5046	0.168833	newly 63 weeks cumul	98 793.	86 394.9	{78 316., 92 371.}	12.5496	0.0597804
19	newly 3 weeks spec	12 889.	8366.35	{7922., 8878.}	35.0892	0.188178	newly 3 weeks cumul	24 953.	16 804.8	{15 918., 17 995.}	32.6542	0.172286	newly 5 weeks spec	14 542.	11 758.8	{11 456., 12 032.}	19.139	0.0923313	newly 5 weeks cumul	54 787.	39 402.1	{38 043., 41 075.}	28.0813	0.143387	newly 58 weeks cumul	96 606.	84 474.1	{79 959., 89 177.}	12.5581	0.058767
20	newly 3 weeks spec	19 368.	17 047.1	{15 726., 18 143.}	11.9831	0.0562127	newly 3 weeks cumul	33 454.	29 881.7	{26 766., 32 528.}	10.7144	0.0505051	newly 5 weeks spec	16 864.	15 571.2	{14 973., 16 185.}	7.74182	0.035163	newly 5 weeks cumul	72 041.	64 800.8	{60 950., 67 059.}	10.0501	0.0462777	newly 63 weeks cumul	97 397.	90 593.7	{81 831., 92 506.}	6.98512	0.0318612
21	newly 3 weeks spec	19 672.	16 214.7	{11 790., 20 446.}	19.4749	0.102995	newly 3 weeks cumul	30 439.	26 516.8	{18 336., 34 393.}	20.2913	0.105622	newly 5 weeks spec	18 543.	15 620.7	{12 740., 18 592.}	16.2646	0.0822862	newly 5 weeks cumul	73 114.	61 882.6	{56 011., 71 483.}	15.3615	0.0747547	newly 64 weeks cumul	98 246.	82 046.5	{74 973., 91 294.}	16.4887	0.0800698
22	newly 3 weeks spec	14 910.	12 509.7	{10 125., 14 119.}	16.0988	0.0803734	newly 3 weeks cumul	31 688.	27 549.2	{23 045., 30 603.}	13.0669	0.0635663	newly 5 weeks spec	14 003.	9895.84	{7324., 12 002.}	29.3306	0.160722	newly 5 weeks cumul	61 831.	49 907.	{40 457., 56 363.}	19.2849	0.0973887	newly 60 weeks cumul	92 206.	67 322.9	{51 579., 78 940.}	26.9865	0.144648
23	newly 3 weeks spec	16 317.	16 902.4	{16 444., 17 353.}	3.6435	0.0154433	newly 3 weeks cumul	34 910.	37 371.3	{35 695., 39 153.}	7.05053	0.0292829	newly 5 weeks spec	13 260.	11 597.6	{11 310., 11 857.}	12.5371	0.0582576	newly 5 weeks cumul	65 072.	64 525.7	{62 779., 66 414.}	2.37849	0.010408	newly 61 weeks cumul	83 725.	83 965.	{80 989., 87 268.}	3.49314	0.0151123
24	newly 3 weeks spec	16 757.	7443.6	{3042., 10 320.}	55.5792	0.413934	newly 3 weeks cumul	25 641.	11 819.2	{4836., 16 388.}	53.905	0.395835	newly 5 weeks spec	20 012.	15 384.5	{10 446., 17 848.}	23.1234	0.144946	newly 5 weeks cumul	69 280.	39 970.9	{22 017., 50 410.}	42.3052	0.281381	newly 63 weeks cumul	98 228.	82 828.7	{59 944., 91 878.}	15.6771	0.0795157
25	newly 3 weeks spec	12 372.	10 562.	{10 206., 11 413.}	14.6296	0.0715133	newly 3 weeks cumul	23 919.	21 382.	{20 476., 23 647.}	10.8156	0.0518623	newly 5 weeks spec	15 457.	12 524.8	{12 485., 12 956.}	18.97	0.0950602	newly 5 weeks cumul	55 191.	46 662.7	{45 994., 49 787.}	15.4523	0.075567	newly 59 weeks cumul	91 517.	82 884.5	{83 191., 86 456.}	9.4327	0.0471019
26	newly 3 weeks spec	12 283.	11 423.2	{11 198., 11 640.}	7.00033	0.0315755	newly 3 weeks cumul	28 429.	28 207.5	{27 658., 28 733.}	1.31211	0.00574004	newly 5 weeks spec	11 730.	9185.8	{8955., 9348.}	21.6897	0.106373	newly 5 weeks cumul	53 475.	48 444.4	{47 699., 48 955.}	9.40735	0.0429334	newly 57 weeks cumul	80 102.	69 514.1	{68 817., 69 745.}	13.46	0.0627871
27	newly 3 weeks spec	12 409.	11 712.4	{11 385., 11 963.}	5.61391	0.025159	newly 3 weeks cumul	28 757.	27 873.1	{27 450., 28 237.}	3.08085	0.0136206	newly 5 weeks spec	11 908.	10 575.7	{9214., 10 968.}	11.1884	0.0523802	newly 5 weeks cumul	53 686.	50 364.6	{48 406., 51 171.}	6.1867	0.0278161	newly 57 weeks cumul	90 968.	82 225.1	{69 325., 85 065.}	9.611	0.0450997
28	newly 3 weeks spec	16 959.	18 644.3	{16 245., 20 566.}	11.5424	0.046648	newly 3 weeks cumul	28 083.	31 933.7	{27 489., 35 588.}	15.3794	0.0612516	newly 5 weeks spec	18 063.	12 727.5	{9821., 15 545.}	29.5384	0.157439	newly 5 weeks cumul	68 202.	63 901.5	{57 078., 71 285.}	8.1145	0.0373134	newly 64 weeks cumul	88 415.	76 587.	{65 386., 91 231.}	14.6408	0.0704729
29	newly 3 weeks spec	12 914.	12 501.6	{10 633., 14 692.}	12.6843	0.0572129	newly 3 weeks cumul	24 569.	24 345.8	{20 205., 28 883.}	13.2862	0.0587014	newly 5 weeks spec	15 388.	12 629.2	{11 387., 13 996.}	17.9282	0.0879525	newly 5 weeks cumul	56 007.	51 345.2	{44 670., 58 955.}	11.5035	0.054335	newly 61 weeks cumul	96 532.	75 906.4	{67 781., 91 215.}	21.3666	0.109046
30	newly 3 weeks spec	13 125.	10 666.8	{6934., 14 771.}	26.6315	0.15771	newly 3 weeks cumul	20 142.	16 271.1	{10 127., 23 317.}	29.0734	0.170697	newly 5 weeks spec	19 690.	17 959.3	{14 537., 19 623.}	8.85582	0.0463604	newly 5 weeks cumul	60 177.	51 473.9	{42 483., 60 276.}	15.4253	0.085381	newly 64 weeks cumul	98 710.	87 197.1	{79 331., 93 568.}	11.6633	0.0551903
31	newly 3 weeks spec	10 370.	10 152.1	{9990., 10 312.}	2.15931	0.00951407	newly 3 weeks cumul	26 082.	26 343.3	{25 975., 26 679.}	1.20528	0.00519479	newly 5 weeks spec	9931.	8884.06	{8725., 9037.}	10.5421	0.0484172	newly 5 weeks cumul	46 868.	45 213.9	{44 782., 45 624.}	3.52921	0.0156149	newly 54 weeks cumul	76 589.	74 542.2	{74 292., 74 801.}	2.67251	0.011766
32	newly 3 weeks spec	12 614.	12 465.2	{10 271., 13 570.}	9.09156	0.0412673	newly 3 weeks cumul	26 998.	28 439.4	{23 287., 31 164.}	12.8369	0.0545911	newly 5 weeks spec	13 001.	8761.71	{7393., 9419.}	32.6074	0.174118	newly 5 weeks cumul	54 036.	48 914.3	{40 849., 52 547.}	9.82615	0.0473359	newly 60 weeks cumul	93 822.	62 193.7	{51 746., 66 286.}	33.711	0.181479
33	newly 3 weeks spec	20 264.	17 380.2	{13 335., 24 359.}	23.5548	0.115279	newly 3 weeks cumul	37 971.	38 754.9	{28 374., 54 896.}	18.4848	0.0767153	newly 5 weeks spec	15 166.	6855.23	{2358., 9358.}	54.7987	0.40866	newly 5 weeks cumul	73 450.	58 145.6	{45 156., 79 280.}	24.4715	0.12702	newly 64 weeks cumul	96 763.	64 062.7	{47 050., 86 387.}	33.7943	0.19168
34	newly 3 weeks spec	15 602.	13 452.5	{13 087., 13 821.}	13.7773	0.0644927	newly 3 weeks cumul	34 185.	31 417.1	{29 897., 32 767.}	8.09674	0.0369429	newly 5 weeks spec	13 645.	8760.89	{8252., 9341.}	35.7941	0.193099	newly 5 weeks cumul	63 916.	52 287.	{51 415., 53 150.}	18.1942	0.087281	newly 60 weeks cumul	89 965.	65 070.1	{64 232., 66 189.}	27.6718	0.140788
35	newly 3 weeks spec	18 683.	17 758.5	{14 032., 20 809.}	12.5959	0.0599154	newly 3 weeks cumul	29 482.	29 483.9	{21 974., 35 139.}	15.0385	0.0688561	newly 5 weeks spec	19 217.	14 872.3	{12 572., 17 616.}	22.6089	0.114803	newly 5 weeks cumul	73 185.	64 573.1	{58 708., 71 763.}	11.7673	0.0562607	newly 64 weeks cumul	94 833.	81 934.2	{74 952., 91 248.}	13.6016	0.065518
36	newly 3 weeks spec	11 929.	8752.31	{6839., 10 984.}	26.63	0.142139	newly 3 weeks cumul	21 930.	16 717.1	{13 423., 20 947.}	23.7705	0.125455	newly 5 weeks spec	15 024.	12 191.9	{8913., 13 909.}	18.8505	0.0980534	newly 5 weeks cumul	52 068.	40 511.2	{31 877., 48 536.}	22.1956	0.114888	newly 60 weeks cumul	97 587.	76 924.9	{52 187., 87 957.}	21.173	0.114365
37	newly 3 weeks spec	15 965.	5314.2	{3172., 7609.}	66.7134	0.506344	newly 3 weeks cumul	25 084.	9221.56	{5391., 13 118.}	63.2373	0.461841	newly 5 weeks spec	18 951.	11 675.9	{8705., 14 322.}	38.3888	0.226438	newly 5 weeks cumul	65 780.	29 713.5	{20 674., 39 307.}	54.8289	0.365141	newly 61 weeks cumul	98 650.	81 668.8	{53 691., 91 727.}	17.2136	0.0936075
38	newly 3 weeks spec	14 686.	12 299.1	{12 074., 12 499.}	16.2531	0.0770718	newly 3 weeks cumul	31 691.	27 926.2	{27 316., 28 452.}	11.8797	0.054975	newly 5 weeks spec	13 535.	11 482.3	{11 318., 11 619.}	15.1656	0.0714531	newly 5 weeks cumul	60 924.	52 237.2	{51 505., 52 812.}	14.2584	0.0668273	newly 58 weeks cumul	87 770.	85 432.6	{82 437., 88 051.}	2.88556	0.0129018
39	newly 3 weeks spec	14 352.	11 774.4	{11 222., 12 752.}	17.9599	0.0864432	newly 3 weeks cumul	28 679.	24 460.5	{23 361., 25 648.}	14.7094	0.0693832	newly 5 weeks spec	15 042.	11 151.7	{10 482., 13 336.}	25.8629	0.132069	newly 5 weeks cumul	60 279.	48 527.9	{46 767., 53 708.}	19.4945	0.0947659	newly 60 weeks cumul	93 593.	70 308.2	{66 608., 87 718.}	24.8788	0.126862
40	newly 3 weeks spec	14 498.	16 801.9	{16 315., 17 357.}	15.8909	0.0639419	newly 3 weeks cumul	31 327.	37 048.	{35 585., 39 005.}	18.2622	0.0725824	newly 5 weeks spec	13 194.	11 545.2	{11 236., 11 844.}	12.4967	0.0580559	newly 5 weeks cumul	59 514.	64 117.4	{62 605., 66 271.}	7.73504	0.0322475	newly 61 weeks cumul	94 658.	83 164.9	{80 972., 87 192.}	12.1417	0.0564592
41	newly 3 weeks spec	13 435.	4489.12	{32., 9275.}	66.9273	0.957232	newly 3 weeks cumul	19 619.	6674.97	{62., 13 703.}	66.6634	0.920791	newly 5 weeks spec	23 349.	11 373.8	{83., 19 432.}	51.2877	0.744634	newly 5 weeks cumul	66 595.	26 693.7	{181., 50 030.}	59.9164	0.828979	newly 64 weeks cumul	95 882.	80 536.5	{56 409., 93 222.}	16.0045	0.12893
42	newly 3 weeks spec	10 315.	10 005.6	{9746., 10 208.}	3.09375	0.0137397	newly 3 weeks cumul	25 161.	24 984.3	{24 479., 25 546.}	1.53201	0.00674068	newly 5 weeks spec	10 541.	9021.46	{8850., 9171.}	14.4155	0.0676436	newly 5 weeks cumul	46 907.	44 111.9	{43 415., 44 817.}	5.95873	0.0267358	newly 55 weeks cumul	87 719.	70 937.5	{70 416., 71 345.}	19.131	0.0922321
43	newly 3 weeks spec	12 045.	12 176.5	{11 222., 13 013.}	5.42233	0.0235354	newly 3 weeks cumul	24 152.	24 909.1	{23 452., 26 180.}	4.95549	0.0211901	newly 5 weeks spec	14 237.	12 047.2	{10 598., 13 559.}	15.3809	0.0753904	newly 5 weeks cumul	52 694.	50 572.8	{46 841., 54 328.}	6.30508	0.0286598	newly 60 weeks cumul	95 113.	76 958.3	{66 882., 87 913.}	19.0875	0.0960706
44	newly 3 weeks spec	16 196.	12 342.5	{11 451., 13 063.}	23.7931	0.118554	newly 3 weeks cumul	32 797.	25 154.3	{23 855., 26 167.}	23.3029	0.115496	newly 5 weeks spec	14 170.	12 283.5	{10 599., 13 565.}	13.3133	0.0646913	newly 5 weeks cumul	64 055.	51 270.9	{47 300., 54 340.}	19.958	0.0974846	newly 60 weeks cumul	95 957.	78 943.	{66 996., 87 950.}	17.7309	0.0884669
45	newly 3 weeks spec	16 795.	15 907.3	{14 229., 17 191.}	6.5329	0.0302162	newly 3 weeks cumul	31 747.	30 153.7	{26 220., 33 400.}	7.94573	0.0366769	newly 5 weeks spec	14 996.	13 972.3	{13 323., 14 737.}	6.84576	0.0310651	newly 5 weeks cumul	65 154.	61 433.4	{56 435., 64 852.}	5.72043	0.0262873	newly 62 weeks cumul	96 985.	84 883.4	{76 864., 90 454.}	12.4778	0.0588443
46	newly 3 weeks spec	15 111.	18 936.1	{16 583., 20 670.}	25.6501	0.0973118	newly 3 weeks cumul	25 292.	31 913.2	{27 640., 35 216.}	26.8903	0.101601	newly 5 weeks spec	17 991.	14 112.5	{10 929., 15 765.}	21.6141	0.10946	newly 5 weeks cumul	63 693.	66 327.3	{57 955., 71 937.}	8.39314	0.0353415	newly 64 weeks cumul	97 957.	82 084.8	{66 133., 91 342.}	16.2032	0.0796046
47	newly 3 weeks spec	13 356.	11 625.8	{11 402., 11 913.}	12.9549	0.060322	newly 3 weeks cumul	27 095.	24 009.3	{23 511., 24 540.}	11.3883	0.0525767	newly 5 weeks spec	14 547.	12 742.8	{12 590., 12 924.}	12.4026	0.0575305	newly 5 weeks cumul	57 180.	50 211.	{49 565., 50 866.}	12.1879	0.0564687	newly 59 weeks cumul	92 154.	85 824.2	{85 624., 86 035.}	6.86874	0.0309054
48	newly 3 weeks spec	14 508.	13 883.1	{12 641., 14 950.}	6.22746	0.0281432	newly 3 weeks cumul	25 267.	24 863.1	{22 182., 27 600.}	6.62924	0.0291927	newly 5 weeks spec	17 877.	15 339.2	{14 383., 15 868.}	14.1958	0.0669073	newly 5 weeks cumul	62 499.	57 309.7	{54 243., 59 907.}	8.34167	0.0381329	newly 62 weeks cumul	95 356.	87 517.4	{83 752., 90 485.}	8.22037	0.0377666
49	newly 3 weeks spec	15 110.	11 913.	{11 362., 12 261.}	21.5657	0.10595	newly 3 weeks cumul	34 829.	30 461.3	{28 980., 31 545.}	13.5217	0.0630328	newly 5 weeks spec	12 499.	6425.79	{5918., 6676.}	48.5896	0.291399	newly 5 weeks cumul	62 570.	46 540.1	{44 771., 46 890.}	25.6627	0.129696	newly 60 weeks cumul	88 106.	54 617.3	{52 792., 54 361.}	38.0095	0.209132
50	newly 3 weeks spec	13 796.	15 646.4	{13 661., 16 856.}	14.5494	0.0579359	newly 3 weeks cumul	28 249.	33 682.2	{28 273., 36 761.}	20.0563	0.0773249	newly 5 weeks spec	14 245.	11 877.2	{11 229., 13 090.}	16.622	0.0797571	newly 5 weeks cumul	58 206.	60 762.2	{56 190., 63 610.}	7.06017	0.0299256	newly 61 weeks cumul	94 140.	82 644.	{80 804., 88 414.}	12.2116	0.0571551
